# Legal Notes for Institutional Workers

**Published:** 1917-10-13

**Authors:** 


### LEGAL NOTES FOR INSTITUTIONAL WORKERS.

### Mistakes in Diagnosis and the Employer's Liability.

Two Workmen's Compensation cases recently reported involved the novel question of the effect of erroneous hospital treatment on a workman's right to compensation. *Bower* v. *Meggitt* and *Mills* v. *Dinnington Co.*, 116 L.T. Rep. 178, 181. In the former of those cases, the immediate result of the accident to the workman proved to be a fracture within the joint of the knee—viz., of the outer condyle of the femur. It was not, however, until an examination under the *x*-rays became available that the precise effect of the injury to the workman's knee was discovered. Previous to that examination he was treated at a hospital on the assumption that no actual fracture had occurred, but that he suffered from arthritis, a natural result of an injury such as that which he had sustained. Was there *novus actus interveniens* in consequence of the mistaken treatment at the hospital—that is to say, had the treatment employed materially modified the condition of affairs? If that could be established, the chain of causation would be broken, and the change of circumstances deprive the workman of his right to compensation. For if a different treatment had originally been resorted to at the hospital, a different state of things would have resulted. The Court held that the onus lay on the employers to prove the change of circumstances, and not upon the workman to prove that his then condition resulted from the accident and not from some intervening cause. In *Mills* v. *Dinnington Main Coal Co., Ltd.*, the other case to which we are now inviting attention, a workman suffering from osteomyelitis, an inflammation of the bone marrow, was treated by the doctor on the supposition that he was suffering from contusion of the soft tissues of the thigh, and possibly of the bone also. As it is an extremely obscure disease and particularly rare among adults, it is not surprising that it required a specialist to diagnose osteomyelitis and septicæmia, rendering indispensable an immediate operation. Owing to this incorrect diagnosis of the workman's real injury, notice of the accident was not given to the employers "as soon as practicable after the happening thereof," as directed by Section 2, Sub-section 1, of the Workmen's Compensation Act, 1906 (6 Edw. 7, c. 58). Inasmuch, however, as the employers were not thereby "prejudiced in their defence," the delay in the notice was regarded by the Court as immaterial. In future instances, where the medical treatment has been based on a mistaken diagnosis, these two cases will probably be found useful as guides to their decision.

